# Pulmonary delivery of cell membrane-derived nanovesicles carrying anti-miRNA155 oligonucleotides ameliorates LPS-induced acute lung injury

**DOI:** 10.1093/rb/rbae092

**Published:** 2024-08-16

**Authors:** Chuanyu Zhuang, Minji Kang, Jihun Oh, Minhyung Lee

**Affiliations:** Department of Bioengineering, College of Engineering, Hanyang University, Seoul 04173, Republic of Korea; Department of Bioengineering, College of Engineering, Hanyang University, Seoul 04173, Republic of Korea; Department of Bioengineering, College of Engineering, Hanyang University, Seoul 04173, Republic of Korea; Department of Bioengineering, College of Engineering, Hanyang University, Seoul 04173, Republic of Korea

**Keywords:** acute lung injury, exosome-mimetic nanovesicle, extracellular vesicles, microRNA155, suppressor of cytokine signaling 1

## Abstract

Acute lung injury (ALI) is a devastating inflammatory disease. MicroRNA155 (miR155) in alveolar macrophages and lung epithelial cells enhances inflammatory reactions by inhibiting the suppressor of cytokine signaling 1 (SOCS1) in ALI. Anti-miR155 oligonucleotide (AMO155) have been suggested as a potential therapeutic reagent for ALI. However, a safe and efficient carrier is required for delivery of AMO155 into the lungs for ALI therapy. In this study, cell membrane-derived nanovesicles (CMNVs) were produced from cell membranes of LA4 mouse lung epithelial cells and evaluated as a carrier of AMO155 into the lungs. For preparation of CMNVs, cell membranes were isolated from LA4 cells and CMNVs were produced by extrusion. Cholesterol-conjugated AMO155 (AMO155c) was loaded into CMNVs and extracellular vesicles (EVs) by sonication. The physical characterization indicated that CMNVs with AMO155c (AMO155c/CMNV) were membrane-structured vesicles with a size of ∼120 nm. The delivery efficiency and therapeutic efficacy of CMNVs were compared with those of EVs or polyethylenimine (25 kDa, PEI25k). The delivery efficiency of AMO155c by CMNVs was similar to that by EVs. As a result, the miR155 levels were reduced by AMO155c/CMNV and AMO155c/EV. AMO155c/CMNV were administered intratracheally into the ALI models. The SOCS1 levels were increased more efficiently by AMO155c/CMNV than by the others, suggesting that miR155 effectively was inhibited by AMO155c/CMNV. In addition, the inflammatory cytokines were reduced more effectively by AMO155c/CMNV than they were by AMO155c/EV and AMO155c/PEI25k, reducing inflammation reactions. The results suggest that CMNVs are a useful carrier of AMO155c in the treatment of ALI.

## Graphical Abstract

**Figure:**
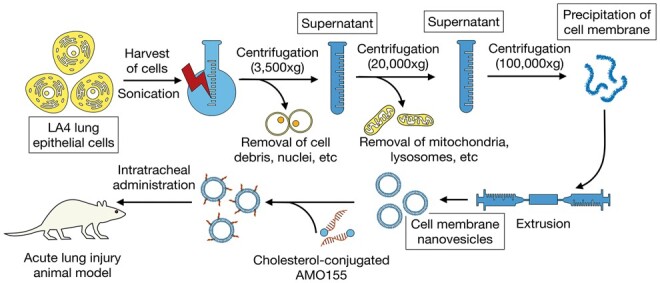

## Introduction

Acute lung injury (ALI) is a devastating inflammatory disease that induces inflammatory reactions, hemolysis and pulmonary microvascular permeability [1]. In the absence of effective treatment, ∼90% of ALI patients will proceed to acute respiratory distress syndrome within 72 h. The clinical treatments of ALI include mechanical ventilation and diuresis for effective oxygen supply and relief of pulmonary edema, respectively [2, 3]. Glucocorticoids were investigated widely for ALI treatment, but they do not reduce the mortality rate of the condition [4, 5]. In addition, excessive use of glucocorticoids may induce side effects such as cardiovascular and cerebrovascular diseases and osteoporosis [6]. Therefore, new therapeutic modalities are needed for effective treatment of ALI.

Therapeutic gene delivery was suggested as an alternative to conventional therapy for ALI [1]. The main focus of gene therapy research has been to reduce the inflammatory response in ALI since the cytokine storm may be deleterious by increasing lung epithelial apoptosis [1]. Anti-inflammatory genes, such as adiponectin (APN) and heme oxygenase-1, were investigated as therapeutic genes in ALI animal models [7–9]. These studies showed that gene therapy might be effective at inducing anti-inflammatory effects. Recently, microRNAs (miRs) were reported to be possible inducers of inflammatory reactions in lungs with ALI [10]. One miR is microRNA155 (miR155), which is present at significantly higher level in ALI than in healthy lungs [11]. miR155 promotes the expression of inducible nitric oxide synthase, matrix metalloproteinase (MMP)-2 and MMP-9 in macrophages [12]. In addition, miR155 facilitates macrophage proliferation and inflammation by inhibiting the suppressor of cytokine signaling 1 (SOCS1) [13]. Since SOCS1 plays an important role in inhibiting nuclear factor-κB (NF-κB)-mediated expression of pro-inflammatory cytokines, the effects of miR155 on SOCS1 expression may increase the inflammatory responses in ALI [14]. Therefore, it has been suggested that miR155 may be a target of ALI therapy [13]. Also, the delivery of anti-miR155 oligonucleotides (AMO155) may be a promising therapeutic strategy for treating ALI.

Extracellular vesicles (EVs) such as exosomes also have been suggested as carriers of small nucleic acids [15]. Prior research has shown that EVs may be an efficient carrier of anti-inflammatory drugs and siRNAs into the lungs [13, 16, 17]. EVs have various sizes ranging from 30 nm to 10 microns. Since they contain various proteins and RNA of their cells of origin, EVs play a role in cell-to-cell signaling [18]. In a previous study, curcumin, a hydrophobic anti-inflammatory drug, was loaded into the EVs and administered into lungs with ALI intratracheally [17]. Curcumin was delivered actively into the lung cells and reduced inflammatory reactions. In another study, EVs were evaluated as carriers of siRNAs into the lungs [16]. The results showed that EVs outperformed liposomes for siRNA delivery into the lungs. Therefore, EVs may be able to carry therapeutic nucleic acids into the lungs. Recently, cell membrane-derived nanovesicles (CMNVs) were suggested as an EV-like nanovesicle. CMNVs may have fewer cellular contents than do EVs [19]. Furthermore, mass production of CMNVs is easier than that of EVs. However, CMNVs have not been evaluated as a carrier of AMOs into the lungs by local intratracheal administration.

In this study, CMNVs were produced using the cell membranes (CM) of LA4 mouse lung epithelial cells and were investigated as a pulmonary delivery carrier of AMO155. Furthermore, the delivery efficiency and therapeutic efficacy of CMNVs were compared with those of EVs from LA4 cells. The results demonstrated that CMNVs may be an efficient carrier of AMO155 into the lungs. Therefore, CMNVs with cholesterol-conjugated AMO155 (AMO155c) may be useful in the treatment of ALI.

## Materials and methods

### Materials

Mouse macrophage cells (RAW 264.7) and lung epithelial cells (LA4) were acquired from the Korea Cell Line Research Foundation (Seoul, Korea). AMO155c, cholesterol-conjugated-scrambled AMO155 (SCR155c) and Cy5-labeled AMO155c (Cy5-AMO155c) were synthesized from Bioneer (Daejeon, Korea) with the following sequences: AMO155c, 5′-ACCCCTATCACAATTAGCATTAA-cholesterol-3'; SCR155c, 5′-TCACAACCTCCTAGAAAGAGTA-cholesterol-3' [20]. The cell culture media [Dulbecco’s modified eagle’s medium (DMEM) and Roswell Park Memorial Institute 1640 (RPMI1640)] and Dulbecco’s phosphate-buffered saline (DPBS) were purchased from Welgene (Seoul, Korea). The PrimeScript RT reagent kit was purchased from Takara Korea Biomedical Inc. (Seoul, Korea). Lipopolysaccharide (LPS), 3-(4,5-Dimethylthiazol-2-yl)-2,5-diphenyltetrazolium Bromide (MTT) and polyethylenimine (branched, 25 kDa, PEI25k) were obtained from Sigma-Aldrich (St Louis, MO, USA). Enzyme-linked immunosorbent assay (ELISA) kits for interleukin-1β (IL-1β) and interleukin-6 (IL-6) were acquired from Invitrogen (Carlsbad, CA, USA). Fetal bovine serum (FBS) and protein assay reagent were acquired from Thermo Fisher (Waltham, MA, USA). Anti-SOCS1 antibody was acquired from Santa Cruz (Dallas, TX, USA). Goat anti-mouse IgG antibody was acquired from Abcam (Cambridge, MA, USA). The Sensifast SYBR LO-ROX kit was acquired from Bioline (London, UK). The ExoEasy Maxi kit and QIAzol lysis reagent were acquired from Qiagen (Valencia, CA, USA).

### Cell culture

RAW264.7 and LA4 cells were cultured in DMEM containing 10% FBS and RPMI1640 medium containing 15% FBS.

### Preparation of CMNVs

The LA4 cells were harvested by centrifugation and were resuspended in 10 ml DPBS and protease inhibitor cocktail. The cells were subjected to ultrasonication for lysis. Next, CM was isolated by triple-speed gradient centrifugation as described previously [7, 21]. The CM pellet was resuspended in 5% glucose and filtered with a 0.22 µm filter. Finally, the CMNVs were prepared by extruding the pellet through 0.2-µm polycarbonate membranes 10 times. The protein concentrations of the CMNVs were analyzed using a protein assay kit.

### Preparation of EVs

The LA4 cells were propagated in RPMI1640 containing 10% EV-depleted FBS for 3 days. Subsequently, the cells were precipitated by centrifugation at 500 ×g for 15 min. Then, the supernatant was applied to an exoEasy Maxi kit for EV isolation.

### Loading AMO155c onto CMNVs or EVs

The AMO155c was loaded onto CMNVs or EVs using a hydrophobic interaction. The cholesterol moiety of AMO155c was integrated into the membranes of CMNVs or EVs by a hydrophobic interaction. For the preparation of AMO155c/CMNV and AMO155c/EV, a fixed amount of AMO155c was mixed in 5% glucose with CMNVs and EVs for 30 min. The mixtures were sonicated (Branson, Danbury, CT, USA) for 10 min (40 kHz, 80 W).

To measure the loading efficiency, Cy5-AMO155c was loaded onto CMNVs or EVs as described above. Unloaded Cy5-AMO155c was removed by ultracentrifugation at 100 000 ×g for 1 h. The supernatants were removed and the pellets were resuspended with 5% glucose solution. The amount of Cy5-AMO155c was quantified using a fluorometer (Molecular Devices, Sunnyvale, CA), with excitation at 650 nm and emission at 670 nm. The loading efficiency was calculated as follows: Loading efficiency (%) = (Amount of Cy5-AMO155c after loading)/(Total amount of Cy5-AMO155c) × 100.

### Intracellular delivery of AMO155c

Intracellular delivery of AMO155c using CMNVs or EVs was evaluated by flow cytometry. MiR155 is overexpressed in the alveolar epithelial cells, macrophages and monocytes, inhibiting the SOCS1 expression [22]. Therefore, the target cells for the delivery should be those types of cells. In the current study, the LA4 cells were used as an *in vitro* model, because the LA4 cells were mouse lung epithelial cells. The LA4 cells were plated on 12-well microassay plates (1 × 10^4^ cells/well) and were propagated in RPMI1640 medium containing 15% FBS for 24 h prior to sample addition. Cy5-AMO155c was loaded onto CMNVs or EVs at various ratios. Cy5-AMO155c/CMNVs and Cy5-AMO155c/EVs in 5% glucose solution were added to the cells. The amount of Cy5-AMO155c was 0.1 µg per well. The delivery assay was performed using flow cytometry as described previously [19]. The intracellular uptake trafficking study was performed with endocytosis inhibitors, as described previously [19, 23, 24].

### Zeta potential and particle size

AMO155c/CMNVs and AMO155c/EVs were prepared at a 1:3 weight ratio in 5% glucose solution. The zeta potentials and particle sizes were measured as described previously [25].

### Transmission electron microscopy

AMO155c/CMNV and AMO155c/EV were prepared at a 1:3 weight ratio. The samples were dropped onto mesh copper grids (Ted Pell, Redding, CA, USA), negative-stained with 2% uranyl acetate and incubated at 37°C overnight. The morphology of the complexes was investigated by transmission electron microscopy (TEM, JEOL, Tokyo, Japan).

### MTT assay

*In vitro* delivery into LA4 cells was performed as described above. The transfected cells were subjected to MTT assays as described previously [25].

### Hemocompatibility assay

Blood was harvested from the tail vein of Sprague Dawley rats and stored in blood collection tubes with EDTA. The red blood cells were isolated as described previously [25]. The RBCs were incubated with 1 µg of PEI25k, CMNVs and EVs. After 30 min of incubation, the samples were mounted on a cover glass and were observed with a light microscope.

### Quantitative real-time reverse transcription-polymerase chain reaction

The miR155 level was evaluated using real-time reverse transcription-polymerase chain reaction (RT-PCR). To evaluate the miR155 level induced by LPS treatment, RAW264.7 cells were used in the RT-PCR study as pro-inflammatory cytokines are induced in the macrophage cells. RAW264.7 cells were plated at 1 × 10^5^ cells/well in 12-well cell-culture plates. For LPS activation, 60 ng of LPS was added to the cells and incubated for 2 h. After incubation, delivery assay was performed with AMO155c/CMNVs, AMO155c/EVs and AMO155c/PEI25k, as described above. Total RNA was prepared from the cells using the QIAzol lysis reagent following the manufacturers’ instructions. RT-PCR was performed as previously described [19, 25]. The miR155 level was normalized by GAPDH. The data were quantitated using the 2−ΔΔCt approach. The primer sequences were previously described [26].

### Ethics statement

All animal experiments were conducted according to the ethical policies and procedures approved by the Institutional Animal Care and Use Committee (IACUC) of Hanyang University, Seoul, Korea (accreditation number: 2022-0135A).

### LPS-induced ALI mouse model

An LPS-induced ALI mouse model was produced with male BALB/c mice (6 weeks of age, 24 g) as described previously [7, 27]. After the LPS challenge, AMO155c/CMNVs, AMO155c/EVs and AMO155c/PEI25k were prepared in 70 µl of 5% glucose solution and administered into the lungs intratracheally. The amount of AMO155c was fixed to 5 µg per mouse. The mice were sacrificed 24 h after administration, and bronchoalveolar lavage (BAL) fluid and lung tissues were harvested for analysis.

### Confocal microscopy

Cy5-AMO155c (5 µg) was complexed with CMNV, EV and PEI25k at their optimal weight ratios in a 5% glucose solution. The complexes were administered to the LPS-induced mice by intratracheal injection. After 24 h, the mice were sacrificed. Their lungs were embedded in O.C.T. compound and cut into 10 µm sections. The slides were fixed in 4% paraformaldehyde for 10 min and washed twice with 0.01% Tween 100 (TBST). Then, the nuclei were stained with 300 nM DAPI for 15 min at room temperature. The slides were observed using a confocal microscope (Leica Microsystems, Wetzlar, Germany).

### Enzyme-linked immunosorbent assays

BAL fluid and lung tissue extracts were prepared as described previously [28]. BAL fluid and lung tissues were analyzed using IL-6 and IL-1β ELISA kits according to the manufacturer’s instructions.

### Hematoxylin and eosin staining

The lung tissues were fixed in 4% paraformaldehyde in 0.1 M phosphate buffer for 24 h, embedded in paraffin and cut into 7 µm sections. Hematoxylin and eosin (H&E) staining was performed as described previously [7].

### Immunohistochemistry

The lung tissue sections were deparaffinized and rehydrated. The antigens were retrieved and blocked with 10% goat serum containing 1% BSA in Tris-buffered saline for 2 h. After blocking, the sections were incubated with mouse anti-SOCS1 primary antibodies or anti-receptors for advanced glycation endproducts (RAGEs) primary antibodies overnight. After being washed with TBST, the sections were treated with Alexa 488-conjugated anti-mouse secondary antibody as described previously [8]. The stained lung sections were observed using a slide scanner (ZEISS, Axio Scan Z1, Oberkochen, Germany). The fluorescence intensity of SOCS1 was quantified using ImageJ software.

### Statistical analysis

The groups were compared for significant differences using one-way ANOVA. *P* values <0.05 were considered statistically significant.

## Results and discussion

### *In vitro* characterization of CMNVs or EVs as carriers of AMO155c

**Figure 1.**
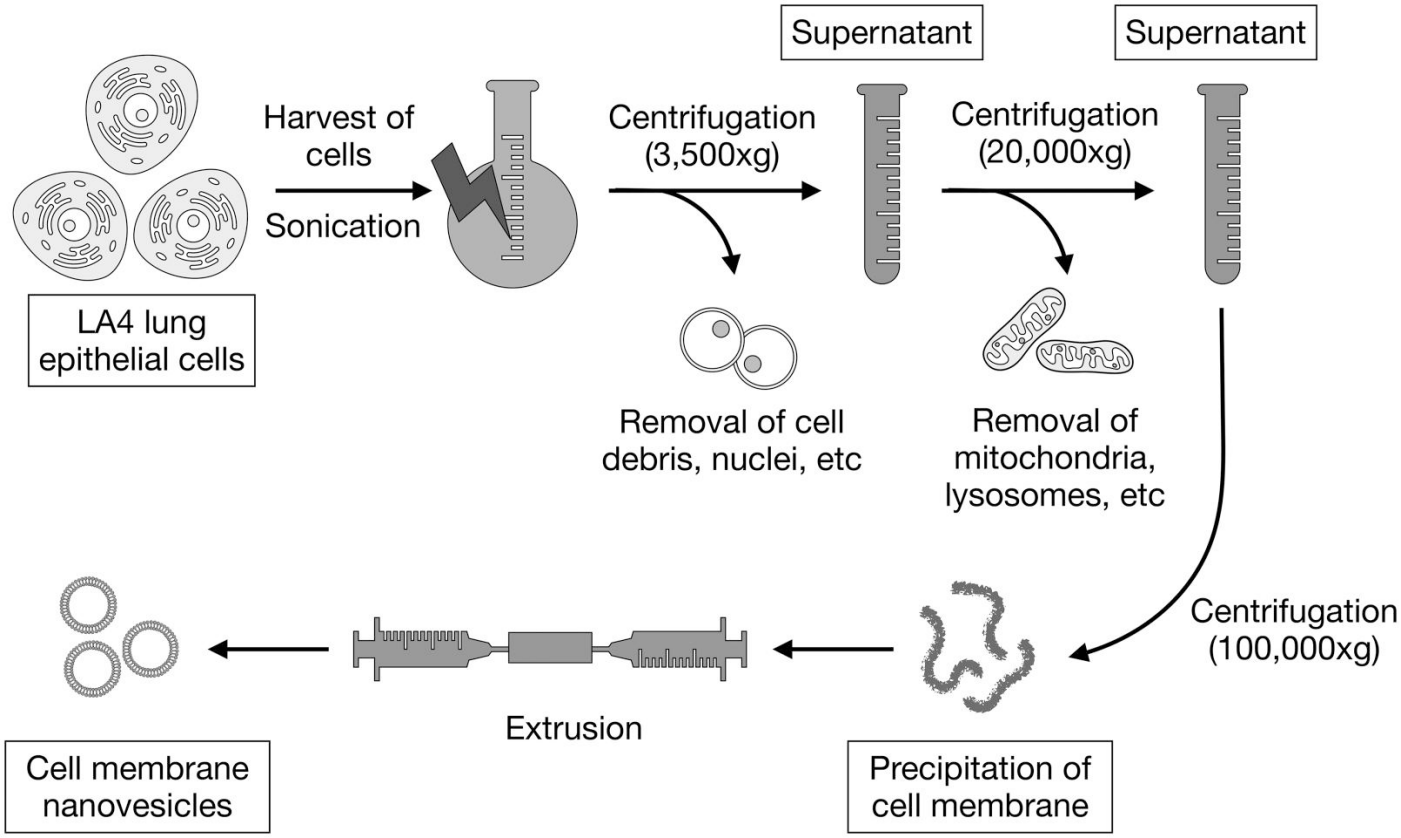
Schematic representation of CMNV preparation.

The CMNVs were prepared using CM isolated from LA4 cells by extrusion (Fig. 1). AMO155c/CMNV and AMO155c/EV were prepared by incubating AMO155c with CMNV and EV in 5% glucose solution. The cholesterol moiety of AMO155c was integrated into the lipid bilayer of the membranes by hydrophobic interaction, as described in a previous study [29]. Since AMO155c was loaded onto the CMNV and EV by hydrophobic interaction, the oligonucleotide moiety of AMO155c may be on the surface of the CMNV and EV with cholesterol moiety integrating into the lipid bilayer. The loading efficiency was 41.2 ± 11.2% of total AMO155c.

**Figure 2.**
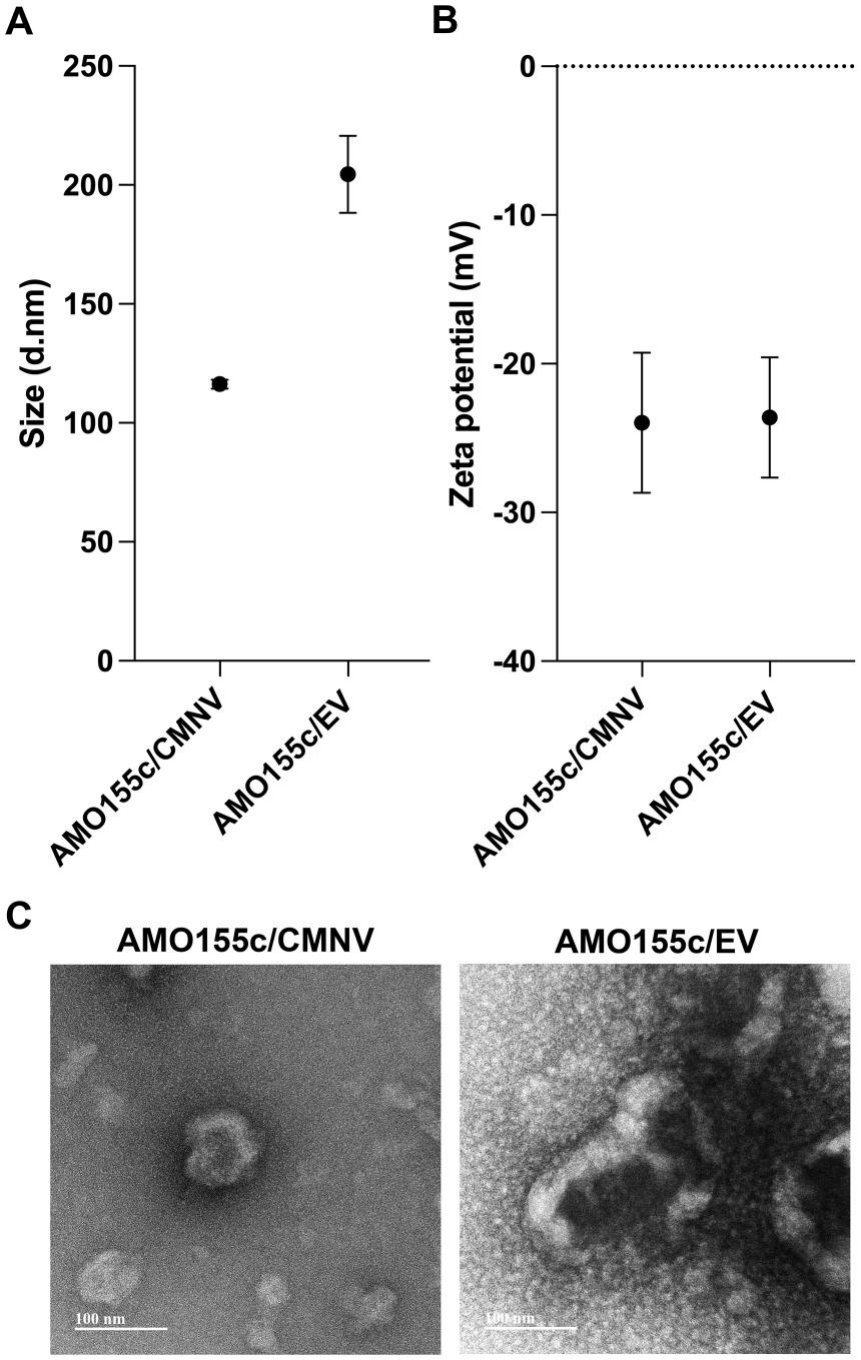
Physical characterization. (**A**) Particle size and (**B**) zeta potential. The sizes and zeta potentials of the micelles were measured by a zetasizer. The data are expressed as the mean ± standard deviation of quadruplicate experiments. (**C**) TEM images. The scale bar is 100 nm.

The particle sizes of AMO155c/CMNV and AMO155c/EV were measured by a zeta-sizer. The size of AMO155c/CMNV was 116.3 ± 1.9 nm, while that of AMO155c/EV was 204.5 ± 16.2 nm (Fig. 2A). Since the CMNVs were produced by extrusion through a filter (molecular cut-off, 200 nm), their molecular size was smaller than that of EVs. However, the surface charges of AMO155c/CMNV and AMO155c/EV were similar at –23.9 ± 4.7 mV and –23.8 ± 4.0, respectively (Fig. 2B). TEM images indicated a spherical morphology of AMO155c/CMNV and AMO155c/EV (Fig. 2C).

**Figure 3.**
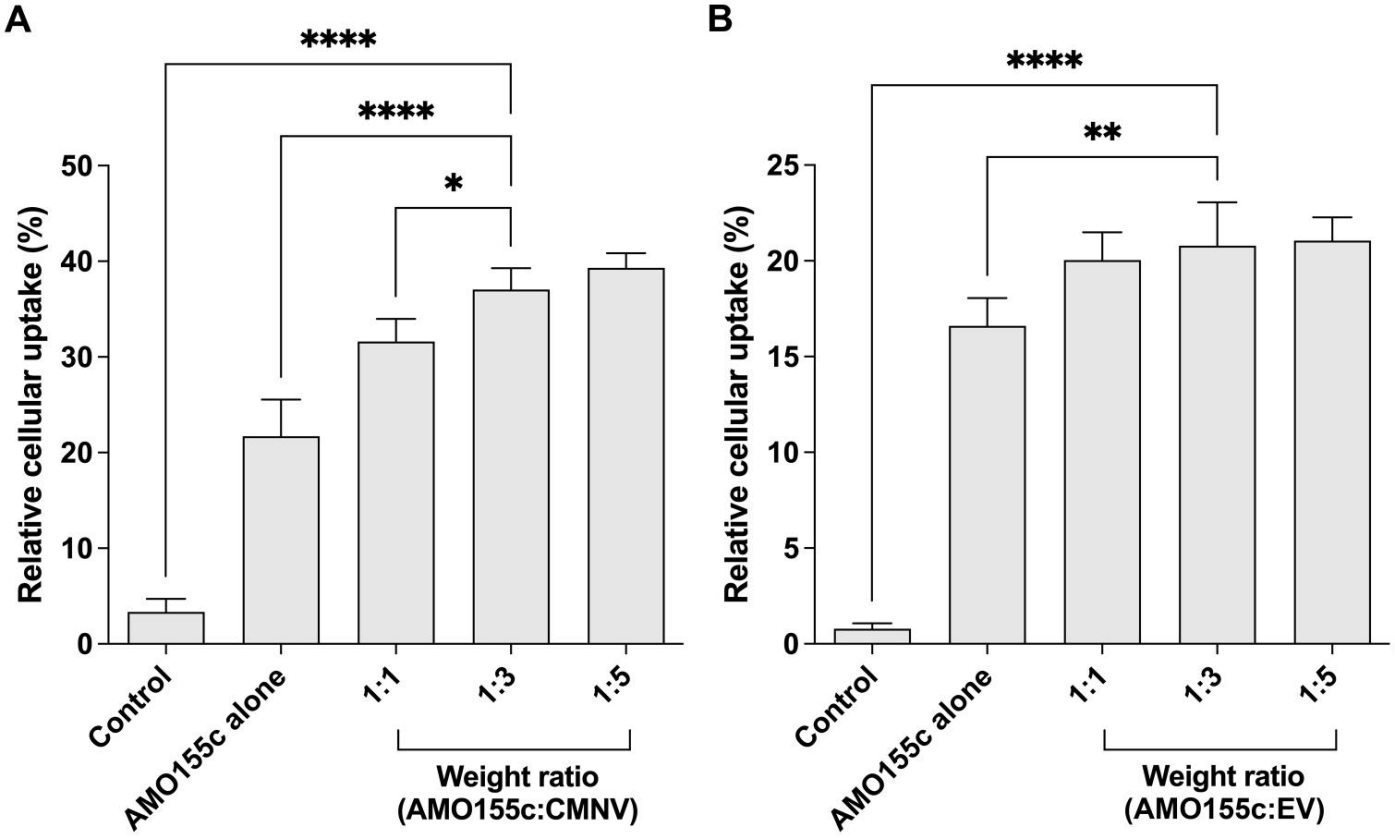
Delivery efficiency of AMO155c using CMNV and EV. Cy5-AMO155c was loaded into CMNV or EV at various weight ratios. AMO155c/CMNV (**A**) and AMO155c/EV (**B**) were transfected into LA4 cells. The delivery efficiency of AMO155c was measured by flow cytometry. The data are expressed as the mean ± standard deviation of quadruplicate experiments. *P < 0.05, **P < 0.01, and ****P < 0.0001.

The delivery efficiencies of CMNVs or EVs were evaluated by delivery assay of AMO155c into LA4 mouse epithelial cells. AMO155c/CMNV and AMO155c/EVs were prepared at different weight ratios. The flow cytometry results indicated that CMNV or EVs had a tendency to increase delivery efficiency up to a 1:3 weight ratio (Fig. 3A and B). Therefore, the ratio was fixed at a 1:3 weight ratio for the following experiments. At a 1:3 weight ratio, the cellular uptake efficiency of AMO155c/CMNV was around 35%, while that of AMO155c/EV around 20% (Fig. 3A and B). This suggests that CMNV might have higher delivery efficiency than EV. The higher delivery efficiency of CMNV might be due to smaller size of AMO155c/CMNV than AMO155c/EV.

### *In vitro* delivery of AMO155c with CMNVs or EVs

**Figure 4.**
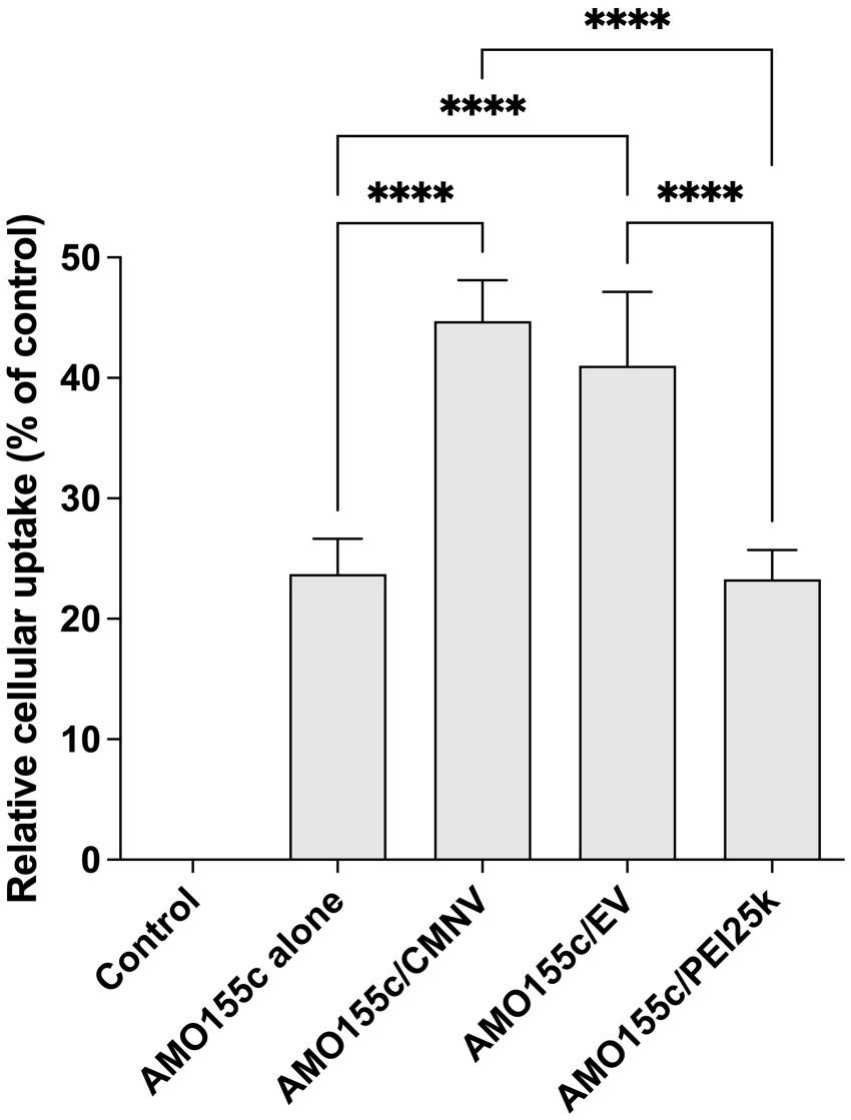
Comparison of AMO155c delivery efficiencies of CMNV, EV and PEI25k. Cy5-AMO155c/CMNV, Cy5-AMO155c/EV and Cy5-AMO155c/PEI25k were prepared as described in the ‘Materials and methods’ Section. The samples were transfected into LA4 cells. The cellular uptake of Cy5-AMO155c was evaluated by flow cytometry. The data are expressed as the mean ± standard deviation of quadruplicate experiments. ****P < 0.0001.

The delivery efficiencies of CMNV and EV were compared with those of PEI25k in the delivery assays. Cy5-AMO155c was delivered into the LA4 cells using CMNV, EV and PEI25k. Flow cytometry was performed to evaluate the intracellular delivery efficiency of the carriers, which was highest in AMO155c in the AMO155c/PEI25k group (Fig. 4). The high delivery efficiency of PEI25k in the *in vitro* delivery has been reported previously in many studies. However, the delivery efficiency of PEI25k into the lungs *in vivo* was not efficient due to capture and clearance by the mucus layer. Due to the high positive charge of AMO155c/PEI25k, the complexes were captured easily by negatively-charged mucins, resulting in clearance in the gastrointestinal tract. The delivery efficiencies of CMNV and EV were higher than those of PEI25k or naked AMO155c (Fig. 4). It was noted that AMO155c may form micelles, increasing its delivery efficiency. However, CMNV and EV had higher delivery efficiencies than did AMO155c.

**Figure 5.**
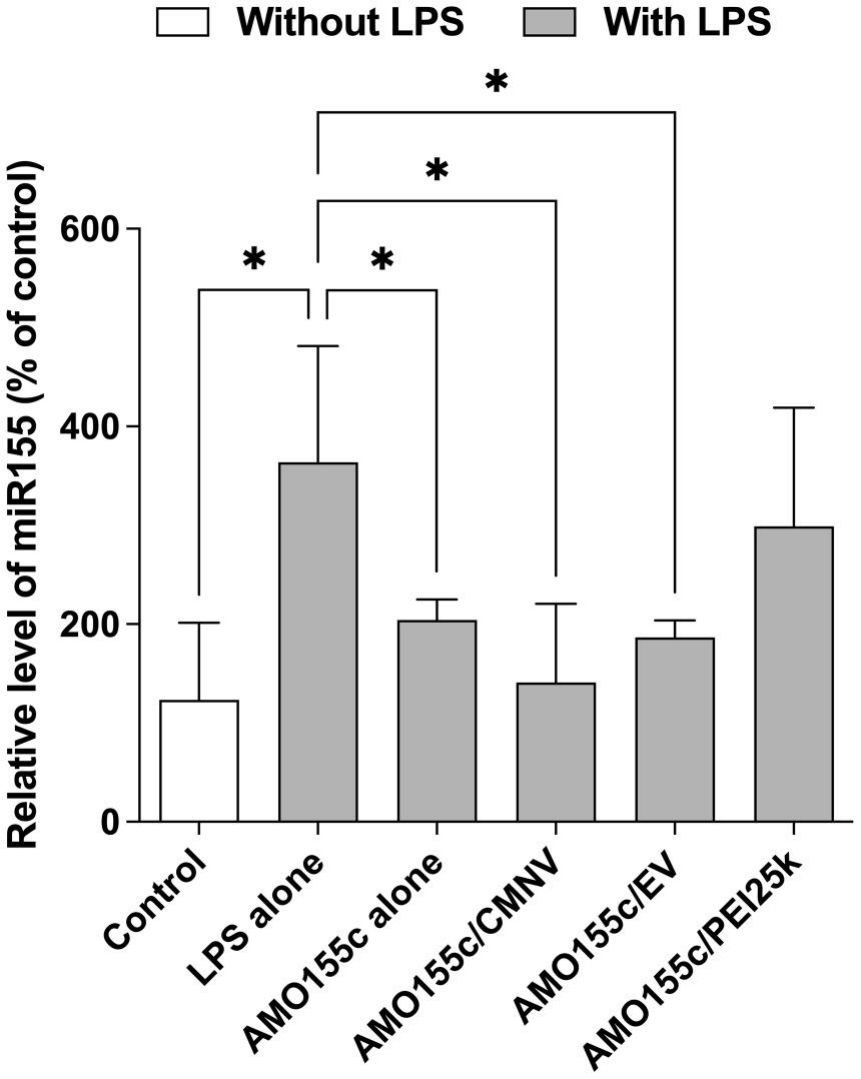
Suppression of miR155 by delivery of AMO155c using CMNV and EV. Naked AMO155c, AMO155c/CMNV, AMO155c/EV or AMO155c/PEI25k were transfected into LPS-activated RAW264.7 cells. After 24 h, the cells were harvested, and the total RNAs were extracted. miR155 was amplified by RT-PCR. The data are expressed as the mean ± standard deviation of quadruplicate experiments. *P < 0.05.

The delivery effect of AMO155c on the miR155 level was evaluated using real-time RT-PCR. The results indicated that the miR155 level was decreased by the addition of naked AMO155c, AMO155c/CMNV or AMO155c/EV (Fig. 5). However, AMO155c/PEI25k did not decrease the miR155 level significantly (Fig. 5). As in Fig. 4, the delivery efficiency of PEI25k was lower than that of AMO155c/CMNV or AMO155c/EV. This low delivery efficiency of PEI25k may contribute to the low efficiency in decreasing the miR155 level. In addition, naked AMO155c had silencing effects, and this might be due to intracellular uptake of significant amount of AMO155c (Fig. 4).

**Figure 6.**
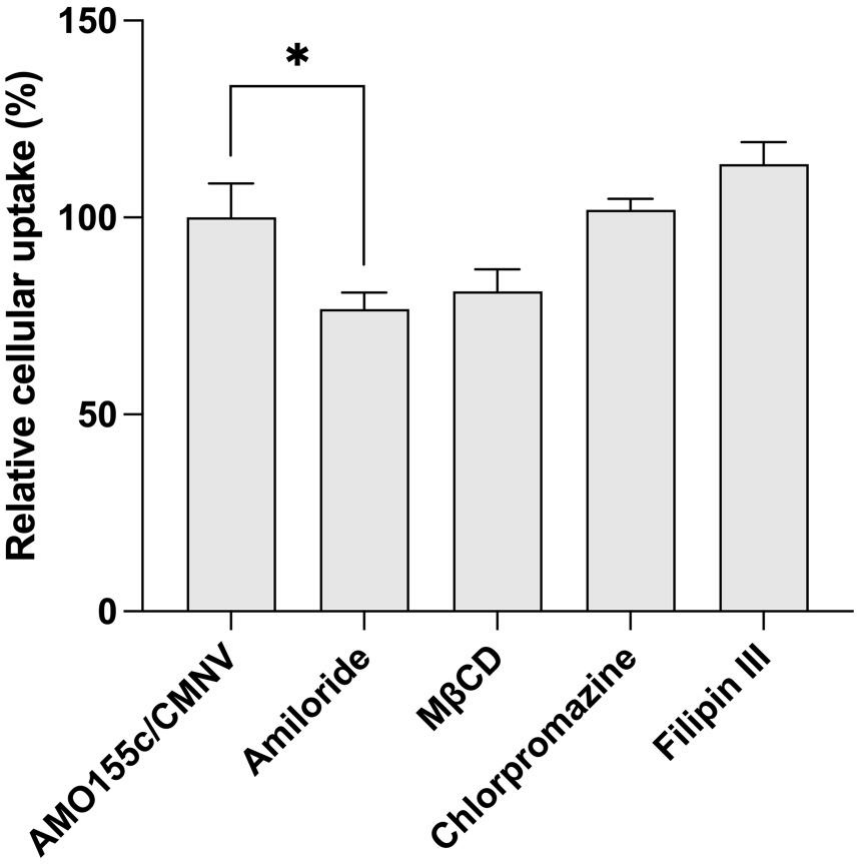
Intracellular trafficking study. The cells were incubated with various inhibitors of intracellular trafficking at 30 min before delivery assay. Cy5-AMO155c/CMNV was prepared and transfected into LA4 cells. The cellular uptake of Cy5-AMO155c was evaluated by flow cytometry. The data are expressed as the mean ± standard deviation of quadruplicate experiments. *P < 0.05.

The intracellular trafficking pathway of the CMNVs was evaluated by delivery assays in the presence of various endocytosis inhibitors (Fig. 6). The results indicated that the cellular uptake was decreased significantly by the addition of amiloride, which is an inhibitor against macropinocytosis (Fig. 6). Methyl-β-cyclodextrin (MβCD) seemed to decrease the endocytosis of AMO155c/CMNV, suggesting that cholesterol-raft-dependent endocytosis may contribute to the endocytosis of AMO155c/CMNV (Fig. 6). However, the decrease by MβCD was not significant compared to that by the control; therefore, further clarification is required. The results also indicate that other inhibitors, such as chlorpromazine and filipin III, did not reduce the cellular uptake (Fig. 6). Therefore, caveolae/raft-mediated endocytosis and clathrin-mediated endocytosis may not be involved in the intracellular trafficking of AMO155c/CMNV.

**Figure 7.**
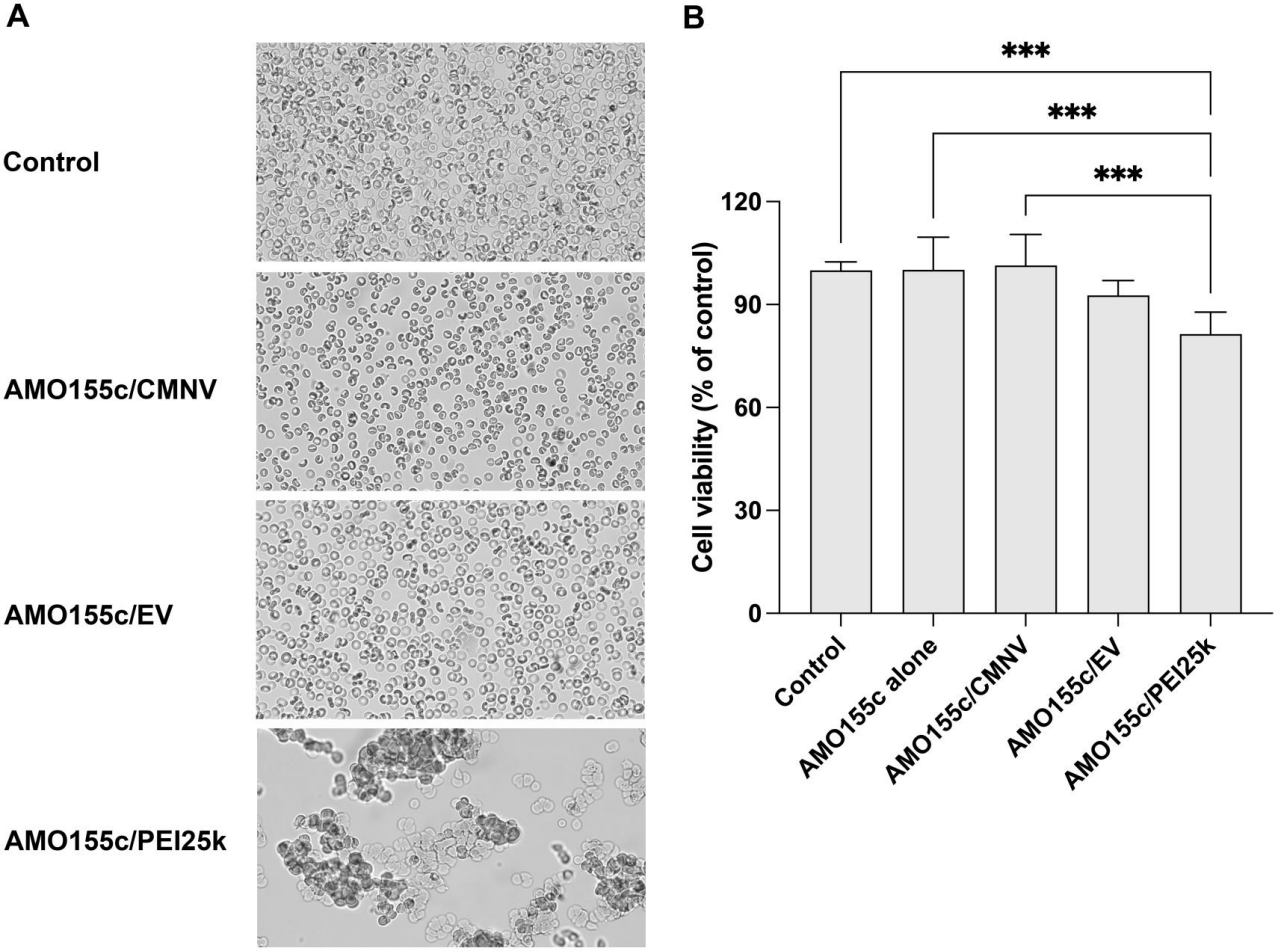
Biocompatibility assay. (**A**) Hemolysis assay. Red blood cells were prepared and incubated with AMO155c/CMNV, AMO155c/EV or AMO155c/PEI25k. The morphology of the cells was investigated by optical microscopy. (**B**) MTT assay. LA4 cells were incubated with AMO155c/CMNV, AMO155c/EV or AMO155c/PEI25k for 24 h. The biocompatibility was evaluated by the MTT assay. The data are expressed as the mean ± standard deviation of sextuplicate experiments. ***P < 0.001.

The biocompatibility of CMNV was evaluated using a hemocompatibility assay and MTT assay. Erythrocytes were incubated with AMO155c/CMNV, AMO155c/EV or AMO155c/PEI25k. The cellular morphology was investigated using light microscopy. The results indicated that the cells in the AMO155c/PEI25k group aggregated and shrunk, suggesting its toxicity (Fig. 7A). In contrast, the cells in the AMO155c/CMNV and AMO155c/EV groups did not show any changes compared to those in the control group (Fig. 7A). This suggests that CMNVs and EVs did not induce significant cellular toxicity. Biocompatibility of AMO155c/PEI25k also was demonstrated by the MTT assay (Fig. 7B). However, AMO155c/CMNV did not induce any remarkable toxicity in the LA4 lung epithelial cells (Fig. 7B).

### Therapeutic effects of AMO155c/CMNV and AMO155c/EV in the ALI animal model

**Figure 8.**
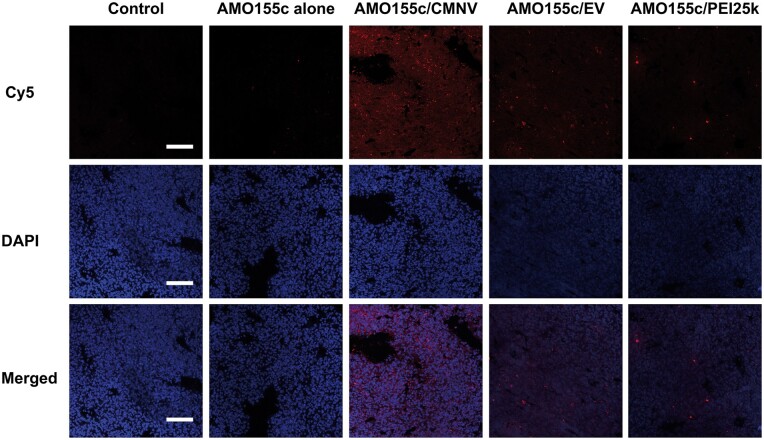
Evaluation of *in vivo* delivery efficiency by confocal microscopy. Naked Cy5-AMO155c, Cy5-AMO155c/CMNV, Cy5-AMO155c/EV, and Cy5-AMO155c/PEI25k were prepared and administered to the ALI animal model by intratracheal instillation. After 24 h, the lung tissues of the animals were harvested and subjected to confocal microscopy. The scale bars indicate 200 µm.

The delivery efficiencies of CMNV and EV were evaluated in the BALB/c mice. Cy5-AMO155c/CMNV and Cy5-AMO155c/EV were administered into the lungs by intratracheal instillation. Also, Cy5-AMO155c alone and Cy5-AMO155c/PEI25k complexes were used as controls. The lungs were harvested, cryo-sectioned and subjected to confocal microscopy. The results indicated that AMO155c/CMNV had higher delivery efficiency than the other samples (Fig. 8).

**Figure 9.**
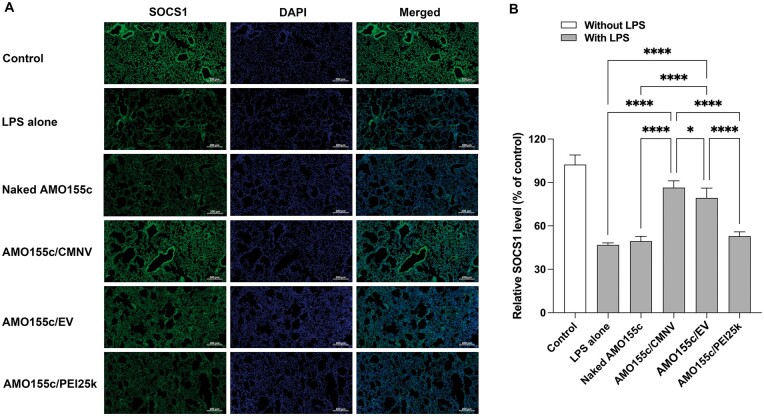
SOCS1 expression after delivery of AMO155c in the ALI animal model. (**A**) Immunohistochemistry. Naked AMO155c, AMO155c/CMNV, AMO155c/EV and AMO155c/PEI25k were prepared and administered to the ALI animal model by intratracheal instillation. After 24 h, the lung tissues of the animals were harvested and subjected to immunohistochemistry with the anti-SOCS1 antibody. The scale bars indicate 200 µm. (**B**) Quantitation of SOCS1 signals. The quantification of the SOCS1 level was achieved using Zeiss Zen software. The data are expressed as mean ± standard deviation of quadruplicate experiments. *P < 0.05 and ****P < 0.0001.

The therapeutic efficacy of AMO155c/CMNV and AMO155c/EV was investigated in an ALI animal model produced by local instillation of LPS into the lungs. The AMO155c/CMNV or AMO155c/EV was administered into the lungs by intratracheal instillation. The therapeutic effects of AMO155c delivery were evaluated by immunohistochemistry of SOCS1, a target gene of miR155 for mRNA degradation [26, 30]. Therefore, the expression of SOCS1 was decreased by the inflammatory reaction. Indeed, the LPS alone group had a lower expression level of SOCS1 than did the control group (Fig. 9A and B). It previously was reported that reduction of SOCS1 expression was involved in the inflammatory reaction in the lungs by inducing secretion of pro-inflammatory cytokines [31]. However, the SOCS1 level was restored by delivery of AMO155c. In particular, AMO155c/CMNV had the highest level of SOCS1 (Fig. 9A and B), followed by that of AMO155c/EV compared to LPS alone, naked AMO155c and AMO155c/PEI25k. However, AMO155c/CMNV more efficiently induced SOCS1 than did AMO155c/EV (Fig. 9A and B).

**Figure 10.**
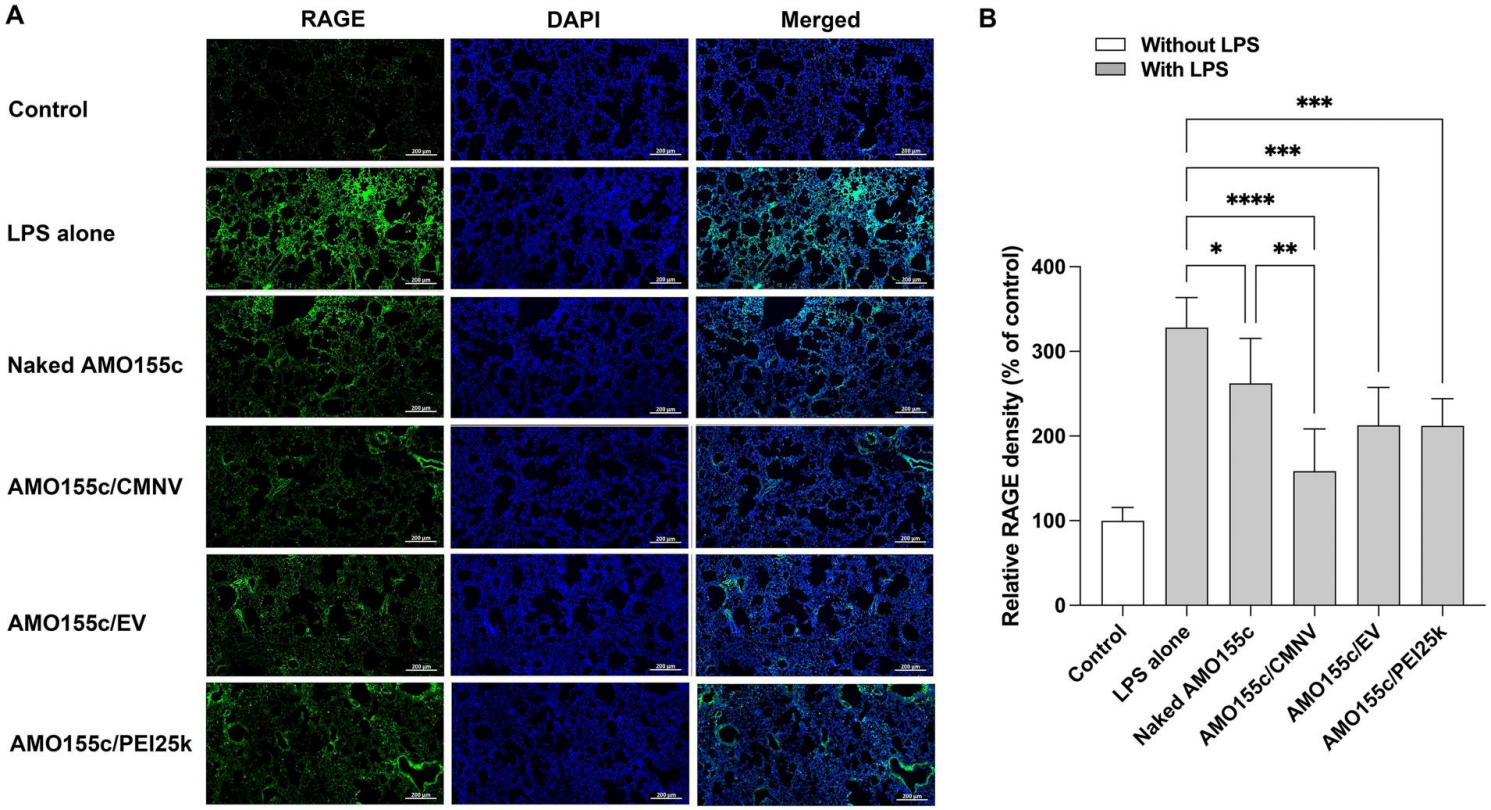
RAGE expression after delivery of AMO155c in the ALI animal model. (**A**) Immunohistochemistry. Naked AMO155c, AMO155c/CMNV, AMO155c/EV and AMO155c/PEI25k were prepared and administered to the ALI animal model by intratracheal instillation. After 24 h, the lung tissues of the animals were harvested and subjected to immunohistochemistry with the anti-RAGE antibody. The scale bars indicate 200 µm. (**B**) Quantitation of RAGE signals. The quantification of the RAGE level was achieved using Zeiss Zen software. The data are expressed as mean ± standard deviation of quadruplicate experiments. *P < 0.05, **P < 0.01, ***P < 0.001 and ****P < 0.0001.

RAGEs were induced in the inflammatory lung tissues, inducing a NF-κB signal cascade. As a result, proinflammatory cytokines are induced in the lungs. In addition, the RAGE expression is also induced by the activation of RAGE-mediated signal cascade. Therefore, the inhibition of RAGE expression has been evaluated as a hallmark of anti-inflammatory effects in the ALI animal models. Therefore, the RAGE levels were measured by immunohistochemistry (Fig. 10). The results indicated that the RAGE level was effectively reduced by AMO155c/CMNV, compared with the other groups.

**Figure 11.**
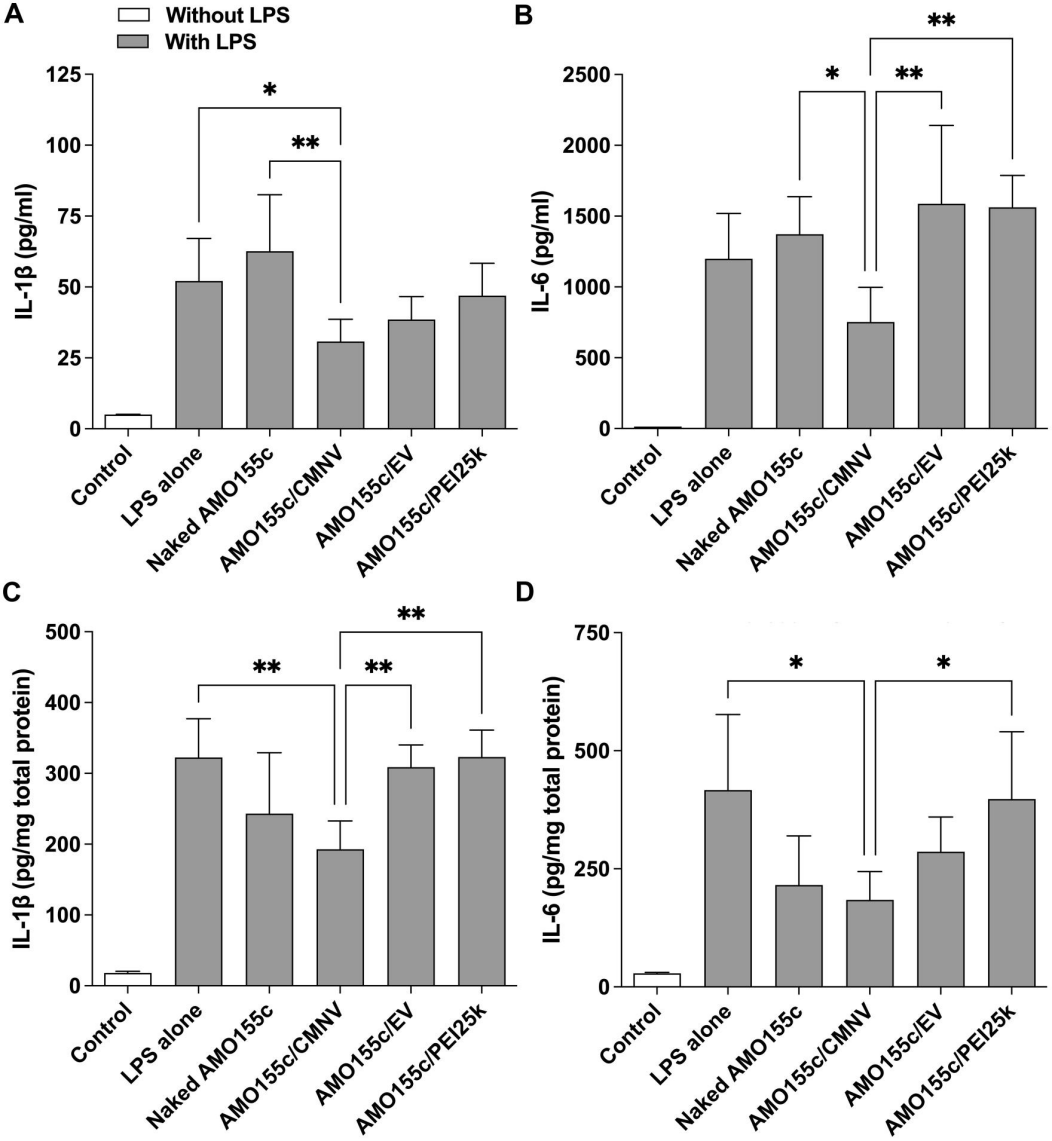
Pro-inflammatory cytokines after delivery of AMO155c in the ALI animal model. Naked AMO155c, AMO155c/CMNV, AMO155c/EV and AMO155c/PEI25k were prepared and administered into the ALI animals by intratracheal instillation. After 24 h, the BAL fluid and lung tissues were harvested and subjected to cytokine ELISAs. (**A**) IL-1β and (**B**) IL-6 in the BAL fluid. (**C**) IL-1β and (**D**) IL-6 in the lung tissues. *P < 0.05 and **P < 0.01.

The pro-inflammatory cytokines may be reduced by delivery of AMO155c since SOCS1 was induced by the delivery of AMO155c. Thus, the cytokine levels in BAL fluids and tissue extracts were measured using ELISA. In the BAL fluids, AMO155c/CMNV effectively decreased IL-1β compared with LPS alone and naked AMO155c (Fig. 11A). Similarly, the IL-6 level was decreased by AMO155c/CMNV compared to that by naked AMO155c, AMO155c/EV and AMO155c/PEI25k (Fig. 11B). These results indicate that improved SOCS1 expression by AMO155c/CMNV effectively reduced the pro-inflammatory cytokine levels. These results were confirmed by ELISAs with tissue extracts. The IL-1β level in the lung extracts was decreased more by AMO155c/CMNV than it was by LPS alone, AMO155c/EV or by AMO155c/PEI25k (Fig. 11C). In addition, the IL-6 level was decreased more efficiently by AMO155c/CMNV than it was by LPS alone or AMO155c/PEI25k (Fig. 11D). These ELISA results from the BAL fluid and tissue extracts indicate that AMO155c/CMNV reduced pro-inflammatory cytokines more efficiently than did the other samples.

In the flow cytometry study, the uptake of AMO155c/CMNV and AMO155c/EV was more efficient than AMO155c alone (Fig. 4). Therefore, it was expected that AMO155c/CMNV and AMO155c/EV might repress miR155 more efficiently than AMO155c alone. However, in Fig. 5, AMO155c/CMNV was not statistically different from AMO155c alone and AMO155c/EV, although the mean value AMO155c/CMNV was smaller than them. However, *in vivo* animal experiments, AMO155c/CMNV repressed miR155 more efficiently than the other samples, increasing the expression of SOCS1 (Fig. 9) and decreasing the cytokine levels (Fig. 10). We speculate that this may be due to the difference of physiological conditions in cell culture and animal experiments. In the previous study, it was suggested that negatively charged nanoparticles may penetrate mucus layer efficiently, reaching epithelial cells [32]. AMO155c/CMNV and AMO155c/EV are negatively charged nano-sized vesicles, while AMO155c did not form stable nanoparticles. Therefore, it is likely that AMO155c/CMNV and AMO155c/EV may have more efficient in the delivery of AMO155c *in vivo* than AMO155c alone.

**Figure 12.**
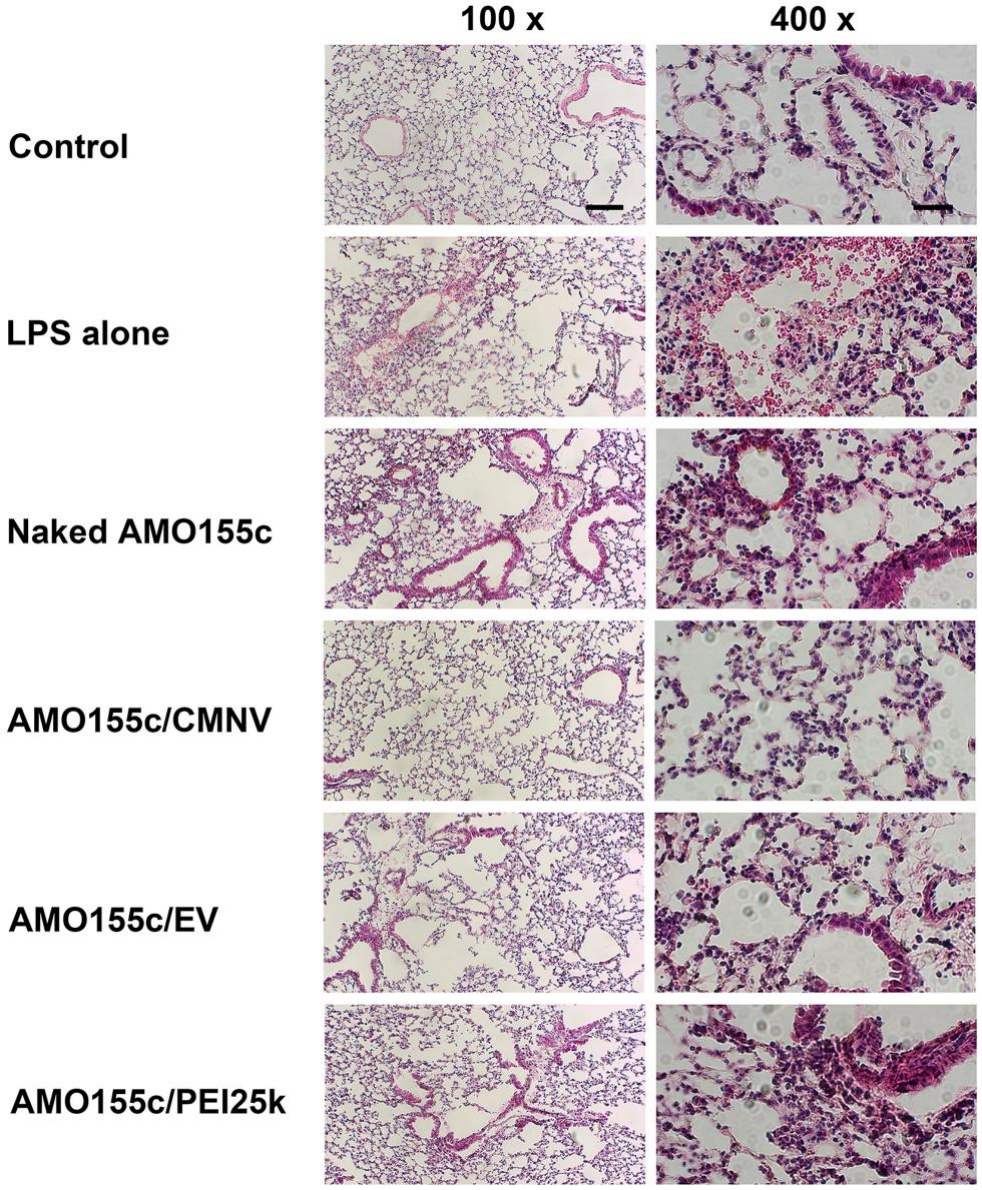
H&E staining. Naked AMO155c, AMO155c/CMNV, AMO155c/EV and AMO155c/PEI25k were prepared and administered into the ALI animals by intratracheal instillation. After 24 h, the lungs were obtained and subjected to H&E staining. The samples were observed by optical microscopy. The scale bars indicate 100 µm in 100× and 25 µm in 400×.

The anti-inflammatory effects of AMO155c/CMNV were investigated using H&E staining (Fig. 12), showing that AMO155c/CMNV, AMO155c/EV and AMO155c/PEI25k reduced the inflammatory reactions in the lungs (Fig. 12). The infiltration of immune cells and hemolysis were induced by the treatment of LPS. In addition, the septa were thickened in the LPS alone samples. Intratracheal administration of AMO155c/CMNV, AMO155c/EV and AMO155c/PEI25k reduced these physiological changes (Fig. 12). The largest decrease was produced by AMO155c/CMNV. The improved anti-inflammatory reactions might be due to increased expression of SOCS1, which was the result of improved AMO155c delivery by CMNVs.

## Conclusions

In this study, CMNVs were investigated as a delivery carrier of AMO155c into the lungs by inhalation. CMNVs were non-toxic to the cells, indicating their biocompatibility. In addition, CMNVs had higher delivery efficiency than did PEI25k, both *in vitro* and *in vivo*. In the *in vivo* evaluation of the ALI animal models, both CMNVs delivered AMO155c into the lungs by inhalation and induced therapeutic effects. Given their biocompatibility and delivery efficiency, CMNV may be a useful carrier of AMO155c in the treatment of ALI.
